# 5-Methylcytosine (m5C) Modification Patterns and Tumor Immune Infiltration Characteristics in Clear Cell Renal Cell Carcinoma

**DOI:** 10.3390/curroncol30010044

**Published:** 2022-12-31

**Authors:** Can Chen, Lin-Yuan Chen, Jie-Xin Zhang, Hua-Guo Xu

**Affiliations:** 1 Department of Laboratory Medicine, The First Affiliated Hospital of Nanjing Medical University, 300 Guang Zhou Road, Nanjing 210029, China; 2 Branch of National Clinical Research Center for Laboratory Medicine, Nanjing 210029, China

**Keywords:** ccRCC, TCGA, m5C methylation, tumor immune microenvironment, immunotherapy

## Abstract

Recently, studies have revealed the prognostic value of 5-methylcytosine (m5C) in clear cell renal cell carcinoma (ccRCC). However, the role of m5C methylation in ccRCC immune infiltration and the immunotherapeutic response remains unknown. Based on the mRNA expressions of 14 m5C regulators, we evaluated the m5C modification patterns of 530 tumor samples from the TCGA-ccRCC database. We used the principal component analysis (PCA) algorithm to construct individual patient m5Cscores to facilitate individual analysis of m5C modification patterns in ccRCC patients. We finally defined three different m5C modification patterns. Different clinical features and immune heterogeneity existed among the three patterns, and their immune infiltration characteristics could correspond to different immune phenotypes, including the immune-inflamed, immune-excluded, and immune-desert phenotype. We designed the m5Cscore calculated by the PCA algorithm to measure individual patients’ m5C modification patterns. The low m5Cscore group presented with a positive prognosis, increased TMB, and immune activation. Additionally, low m5Cscore patients showed an increased response to immune checkpoint inhibitors. We further the value of the m5Cscore in predicting OS verified in four other tumor cohorts. Our findings revealed that m5C methylation modifications are essential in regulating ccRCC immune infiltration. Assessing single ccRCC patients’ m5C modification patterns can fully improve our comprehension of tumor immune characteristics and be used to provide effective personalized immunotherapy strategies for clinical use.

## 1. Introduction

RNA modification has become one of the most cutting-edge and hottest research directions in the life sciences [1,2]. Common RNA modifications include N6-methyladenosine (m6A), N1-methyladenosine (m1A), and 5-methylcytosine (m5C) modifications [3]. In particular, m5C modification occurs in almost all types of RNA, including mRNA and noncoding RNA; the function of the modification varies across RNA isoforms [4]. In tRNA, m5C ensures translation accuracy by regulating RNA structure and stability [5]; in contrast, in rRNA, the loss of m5C terminates the codon translation process [6]. m5C methylation is dynamic and reversible, modulated by a combination of methyltransferases (writers), demethylases (erasers), and binding proteins (readers) [7,8]. Under the action of methyltransferases such as *NSUN1-7* and *DNMT2*, S-adenosylmethionine (SAM) acts as a methyl donor to create 5-methylcytosine [9,10]. m5C methylates RNA functions upon combining with binding proteins, such as *ALYREF* and *YBX1* [9,11]. Demethylases such as *TET2* and *TET3* are used to terminate and remove the above process [12,13]. 

Immune checkpoint inhibitors (ICIs) are currently the most widely used immunotherapy strategies in clinical practice [14,15,16]. Pembrolizumab (PD-1 inhibitor) in combination with axitinib is a first-line treatment for advanced renal cell carcinoma [17]. The tumor immune microenvironment (TME) and its immune characteristics largely influence the clinical efficiency of ICIs [18,19,20]. Based on tumor resistance to PD-L1/PD-1 therapy, Chen et al. classified tumors into three different immune phenotypes, including the immune-inflamed, immune-excluded, and immune-desert phenotypes. The immune-inflamed phenotype is defined as a tumor parenchyma containing numerous CD4 and CD8^+^ T cells with myeloid cells and monocytes. The patients with this phenotype are thus the most clinically responsive to treatment with ICIs. The immune-excluded phenotype is characterized by the existence of numerous immune cells in the surrounding stroma of the tumor. These cells fail to penetrate the matrix to enter the parenchyma and exert an antitumor response. The immune-desert phenotype lacks T-cell expression, particularly that of CD8+ T cells. As a result, these tumors lack an immune response and do not respond to ICIs [19]. Therefore, an adequate understanding of the TME characteristics of various tumor patients is clinically valuable in understanding the patient response to immunotherapy and in developing individualized immunotherapy strategies [21,22]. 

Recently, a correlation between m5C regulators and tumor development and its immune cell infiltration has been found [11,23,24,25]. Chen et al. revealed that m5C RNA modification regulates the RNA upregulation of oncogenes with hypermethylated m5C sites in human urothelial carcinoma of the bladder [11]. Schoeler et al. found that *TET2* and *TET3* are expressed during B-cell progression and terminal differentiation, with both directing the transformation of germinal center B cells into antibody-secreting plasma cells. This indicated that *TET* exerts a vital influence on germinal center expansion, plasma cell differentiation, and antibody production [23]. Miao et al. demonstrated that a lack of NSUN2 repressed vascular chemotaxis to T cells, when T-cell infiltration was reduced in abdominal aortic aneurysms, and the vascular inflammatory response was diminished [24]. Xu et al. found that *TET2* controls chemokine and *PD-L1* expression, lymphocyte infiltration, and cancer immunity by mediating the IFNγ–JAK–STAT–TET pathway. The absence of TET activity diminished the expression of TH1-type chemokines and tumor-infiltrating lymphocytes, thereby allowing an evasion of antitumor immunity and resistance to anti-PD-L1 therapy [25]. The limitations of the experimental conditions have allowed these researchers to focus on only one or two m5C genes and cell types. In contrast, antitumor immunity is characterized by the coordinated action of diverse cells and cytokines, and m5C methylation in cancer involves three different m5C regulators [8]. Therefore, thorough assessment of the spectrum of tumor immune cell infiltration regulated by multiple m5C regulators might facilitate insight into the role of m5C methylation in TME regulation.

In this study, we collated expression and mutational information on m5C regulators from The Cancer Genome Atlas (TCGA) and the International Cancer Genome Consortium (ICGC) databases, as well as patient clinical data, to facilitate thorough evaluation of m5C modification patterns and immune signatures in ccRCC. We defined three m5C modification patterns and found that they have different prognostic and immunological profiles. This pointed to a key function of m5C modifications in shaping the ccRCC TME. We then developed a scoring system based on the spectrum of m5C regulators to quantify m5C modification patterns in individual ccRCC patients and to measure the efficacy of immunotherapy.

## 2. Materials and Methods

### 2.1. ccRCC Datasets and Preprocessing

We downloaded 535 patient and 72 normal samples of m5C regulator expression data, annotated the clinical information and somatic mutation data for 336 patients from the TCGA-ccRCC database, and removed tumor patients with duplicate IDs. We downloaded information on the TCGA-ccRCC database from the GDC website (https://portal.gdc.cancer.gov/ (accessed on 6 December 2020)) [26]. We obtained additional m5c gene expression data from the ICGC database (https://icgc.org/ (accessed on 6 December 2020)), which included a total of 91 tumor and 45 normal samples. We used the “SVA” package to address the batch effects present in both datasets [27]. 

### 2.2. Unsupervised Clustering of m5C Regulators

We identified 14 m5C regulators from two databases. Based on 14 gene expressions, we used the “ConsensusClusterPlus” package to run unsupervised cluster analysis on the tumor samples and to determine the number of specified clusters. We repeated the above steps 1000 times to guarantee the consistency of classification [28].

### 2.3. Estimation of ccRCC Immune Cell Infiltration 

We designed single-sample gene set enrichment analysis (ssGSEA) for calculating the enrichment fraction for each gene set in each ccRCC sample [29,30]. We used the ESTIMATE algorithm with expression data to calculate stromal and immune cell content to evaluate tumor purity [31]. We applied the CIBERSORT deconvolution algorithm to quantify the relevance and significance among 22 immune cells and 14 m5C regulators [32,33].

### 2.4. Differentially Expressed Genes (DEGs) between m5C Clusters

We filtered the DEGs between the 3 m5C modification patterns using the empirical Bayesian approach in the “limma” package [34]. The adjusted *p*-values of these DEGs were less than 0.05.

### 2.5. Gene Set Variation Analysis (GSVA)

GSVA is a nonparametric, unsupervised analytical method that facilitates the exploration of differences in bio-processes between different m5C clusters [35]. We used the “c2.cp.kegg.v6.2.-symbols” gene set downloaded from MSigDB database for GSVA. 

### 2.6. m5C Genetic Signature Generation

We adapted a score system for accurately characterizing individual ccRCC patient patterns of m5C modifications, termed the m5Cscore. We applied univariate Cox regression analysis to the previously extracted DEGs with overlap among the m5C clusters to screen for prognosis-related DEGs. We performed PCA on these prognosis-associated DEGs, and we used PC1 and PC2 as feature scores to construct the m5C gene signature [36,37,38]: m5Cscore = Σ (PC1i + PC2i), where I is the expression of the prognosis-related DEGs between the m5C clusters. 

### 2.7. IPS Analysis

The immunophenoscore (IPS) can serve to predict patient reactions to immune checkpoint inhibitors, quantify the determinants of tumor immunogenicity, and characterize the intratumoral immune landscape. IPSs are distributed between 0 and 10 where the higher the score, the higher the immunogenicity [29]. We obtained the IPS results from The Cancer Immunome Atlas (TCIA) (https://tcia.at/home (accessed on 6 December 2020)).

### 2.8. Statistical Analysis

We performed Spearman correlation analysis to compute the correlation coefficient between m5C regulator expressions and immune cells. We used the Wilcoxon test for analyzing the differences between the two groups. We performed Kruskal–Wallis and one-way ANOVA tests for analyzing the differences among three and more groups. We employed the “survminer” package for determining the optimal cut-off values and dichotomizing patients. We used the Kaplan–Meier method for plotting survival profiles, and we employed log-rank tests for determining statistical significance. We applied the “maftools” package for analyzing the mutations between the different groups. We regarded *p* < 0.05 as statistically significant. We processed data with R 4.0.3 software.

## 3. Results

### 3.1. Somatic Gene Mutation and Expression Patterns of m5C Regulators in ccRCC

**Figure 1:**
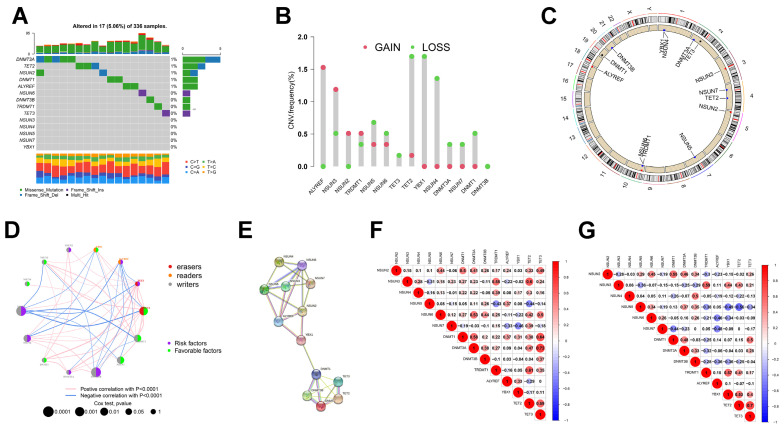
Landscape of genetic and expression variations of m5C regulators in ccRCC. (**A**) Mutation frequency of 14 m5C regulators in 336 ccRCC patients. (**B**) CNV variation frequency of m5C regulators in TCGA-ccRCC cohort. (**C**) Location of CNV alteration of m5C regulators on 23 chromosomes. (**D**) Interaction between m5C regulators in ccRCC. (**E**) PPI network of m5C regulators. (**F**) Spearman correlation analysis of m5C regulators in TCGA cohort. (**G**) Spearman correlation analysis of m5C regulators in ICGC cohort.

We identified 14 m5C regulators in this study. We first analyzed these 14 m5C regulators in ccRCC for copy number variation (CNV) and the occurrence of somatic mutations. As shown in Figure 1A, only 17 (5.06%) of the 336 ccRCC samples showed mutations in the m5C regulators, which is a low mutation frequency. We then found widespread CNV alterations in the m5C regulators, mostly concentrated in copy number deletions, whereas *NSUN2*, *NSUN3*, *ALYREF*, and *TRDMT1* showed a high frequency of CNV amplification (Figure 1B). m5C regulator CNV alterations in chromosomal location are shown in Figure 1C.

Subsequently, we characterized the mRNA expression profiles of the 14 m5C regulators in ccRCC and found that most m5C regulators were significantly differentially expressed (Table S1). The TCGA database showed that all genes except *TRDMT1* are aberrantly expressed in ccRCC. The expressions of *NSUN3*, *NSUN4* and *NSUN7* were lower in tumors relative to normal tissue, whereas the expressions of the remaining genes were increased in tumors (*p* < 0.05). In contrast, the ICGC database revealed no significant differences in *NSUN3*, *DNMT3B*, or *TRDMT1* expressions between the two tissues, and the rest were consistent with the TCGA database. We further analyzed the m5C regulator protein expression levels within both tissues. The protein and mRNA expression levels of the m5C regulators remained consistent (Figure S1A). Moreover, we observed an association between genetic and expression variation in the m5C regulators, with genetic variation affecting m5C regulator expression. The expression of m5C regulators with the deletion of CNV was enhanced in ccRCC in comparison with normal tissues (e.g., *NSUN6* and *DNMT1*) and vice versa (e.g., *NSUN3* and *TRDMT1*) (Figure 1B and Table S1). These findings demonstrated that m5C regulators’ genetic and expression variations are highly heterogeneous between ccRCC and normal tissues, implying that imbalances in m5C regulator expressions substantially contribute to ccRCC development.

Because the role of m5C modifications rely on the coordination between genes, we attempted to elucidate the correlation between the 14 m5C regulators. First, we plotted mRNA and protein reticulation to investigate the interactions among m5C regulators (Figure 1D,E). The results of our analysis of the String database suggested that m5C regulators frequently interact with each other in the protein–protein interaction (PPI) network and that NSUN6 appears to be a core gene of the m5C regulators (Figure 1E). However, no further analysis revealed a correlation in expression between NSUN6 and other regulators. In the TCGA-ccRCC cohort, we observed a positive link between *TET3* and ten regulators, in particular *DTMT1* and *DTMT3A*. Conversely, *NSUN5* negatively interacted with six regulators, notably *TRDMT1* and *TET2* (Figure 1F). This implied that *TET3* and *NSUN5* might be essential elements of the m5C regulators that influence the onset and development of ccRCC. Unlike in the ICGC cohort, *TET2* showed stronger positive correlations with other genes, particularly *YBX1* and *TET3* (Figure 1G). The trends among the m5C regulators in the TCGA and ICGC databases were roughly consistent in a broad sense. The above findings indicated that not only do m5C regulators in the same functional class show significant correlations in expression but also between different classes.

Next, we investigated the prognostic efficacy of the m5C regulators in ccRCC (Figure S1B). The results of the univariate Cox regression models uncovered the predictive value of the 14 m5C regulators for ccRCC patients (Table S2). *NSUN2*, *NSUN5*, *NSUN6*, *DNMT3A*, *DNMT3B*, *ALYREF*, and *TET3* acted as tumor risk factors, and overexpression of these genes led to a poorer prognosis for ccRCC patients. In contrast, the expressions of *NSUN3*, *NSUN4*, *NSUN7*, *TRDMT1*, *DNMT1*, *YBX1,* and *TET2* were low in tumors and had a tumor-suppressive effect. The m5C regulator network diagram provides a more visual representation of the linkages and interactions between m5C regulators and their prognostic performance (Figure 1D).

### 3.2. m5C Modification Patterns Mediated by 14 m5C Regulators

**Figure 2:**
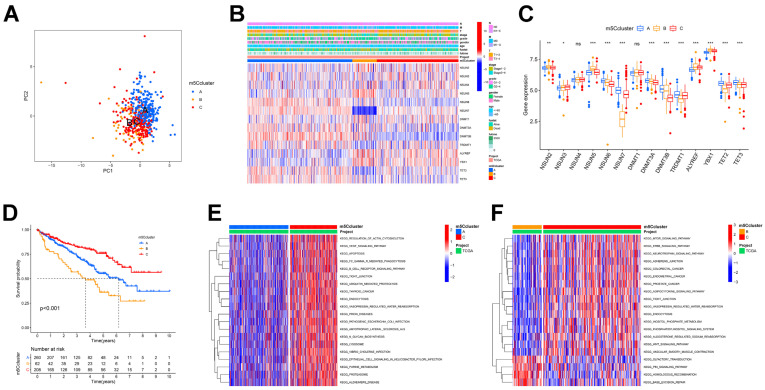
m5C clusters and corresponding biological features in ccRCC. (**A**) PCA for e transcriptome profiles of three m5C clusters. (**B**) Unsupervised clustering of 14 m5C regulators in TCGA-ccRCC cohorts. (**C**) mRNA expression distributions of 14 m5C regulators in three m5C cluster. Upper and lower ends of boxes represent interquartile range of values. (**D**) Kaplan–Meier curves for three m5C modification patterns. (**E**,**F**) GSVA showing activation states of biological pathways in distinct m5C clusters. (**E**) cluster A vs. cluster C; (**F**) cluster B vs. cluster C. * *p* < 0.05; ** *p* < 0.01; *** *p* < 0.001; ns is not significant.

Following constructing the expression profiles of m5C regulators in the TCGA-ccRCC cohort, we ran an unsupervised cluster analysis on these data with the “ConsensusClusterPlus” package to identify three different m5C modification patterns, called m5C clusters A-C. Of these, patterns A, B, and C included 260, 62, and 208 cases, respectively (Figure 2A and Figure S2A). The heat map showed that ccRCC patients with the three modification patterns have different clinical characteristics, with patients in cluster C having significantly better clinical staging than the other two clusters (Figure 2B). Next, we characterized the expression of the 14 regulators in different clusters. Of the 14 regulators, 12 significantly differed between three clusters, with 7 genes being more expressed in cluster A than in the other two clusters, the remaining 4 genes being more expressed in cluster B, and only *NSUN3* being the most expressed in cluster C (Figure 2C). The results of prognostic analysis also demonstrated that patients in cluster C displayed the longest overall survival and the most positive prognosis, next to cluster A (*p* < 0.001, Figure 2D). Subsequently, we ran GSVA and investigated the biological processes among the different m5C clusters. Figure 2E,F show that many cancer activation pathways, stromal activation, and immune activation were significantly enriched in m5Ccluster-C, including the mTOR and ERBB signaling pathways, the adherens and tight junctions, and the BCR signaling pathway. The m5Ccluster-B showed a significant enrichment in genetic information processing related signaling pathways, including homologous recombination and base excision repair. In contrast, cluster A exhibited enrichment in the immunosuppressive pathways.

### 3.3. Analysis of Immune Infiltration in Different m5C Clusters

**Figure 3:**
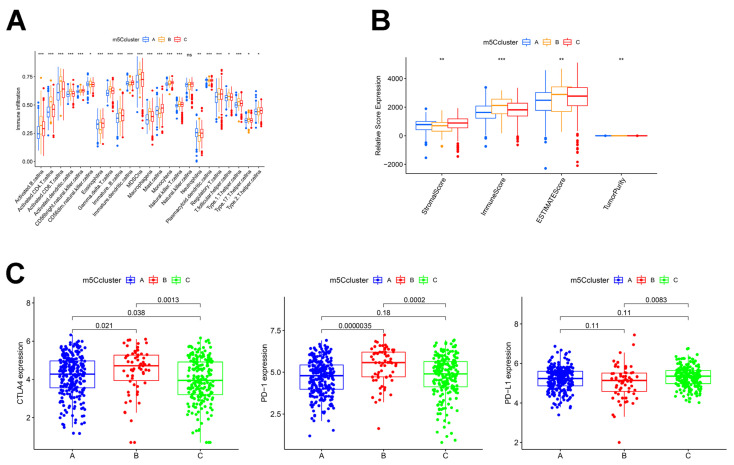
TME cell infiltration characteristics and immune checkpoints in distinct m5C modification patterns. (**A**) Abundance of each immune cell in three m5C modification patterns. (**B**) Box plot indicates differences in immune score, stromal score, estimate score, and tumor purity between different m5C modification patterns. (**C**) RNA expression levels of CTLA-4, PD1, and PD-L1 in distinct m5C clusters. * *p* < 0.05; ** *p* < 0.01; *** *p* < 0.001; ns is not significant.

To better characterize the immunological profile of the m5C modification pattern, we used two different methods to quantify tumor-infiltrating immune cells: ssGSEA and ESTIMATE methods. First, the ssGSEA results showed that m5Ccluster-B was rich in numerous immune cell infiltrates including B cells, T cells, activated dendritic cells, natural killer cells, MDSC, macrophages, monocytes, and others (Figure 3A). From the ESTIMATE analysis, we also found that cluster B had a much higher immune component than the other two groups (Figure 3B). Nevertheless, no matched survival benefit was demonstrated for this m5C modification pattern (Figure 2D). As a result, we concluded that the TME immune infiltration in the three m5C modification patterns is significantly different. From a previous study [19], we hypothesized that m5Ccluster-B patients may be the immune-excluded phenotype, which is characterized by the presence of many immune cells in the tumor stroma rather than parenchyma and the inability to exert immune action. We categorized cluster C as the immune-inflamed phenotype, which is characterized by adaptive immune cells with monocytes infiltration and immune activation. We categorized cluster A as the immune-desert phenotype, in which immune cells, particularly T cells, are less expressed and show immune suppression. The results of the GSVA also confirmed our hypothesis that cluster A was strongly linked to immunosuppression, whereas cluster C was associated with immune and matrix activation (Figure 2E,F). Immediately afterward, we characterized the expressions of the common immune checkpoints in these clusters. As shown in Figure 3C, PD-1 and CTLA-4 expressions were highest in cluster B (*p* < 0.05). This correlated with the high T-cell infiltration in cluster B, which also verified why the cluster B patients had the worst prognosis (Figure 2D).

### 3.4. Establishment of m5C Gene Signature and Functional Annotation

**Figure 4:**
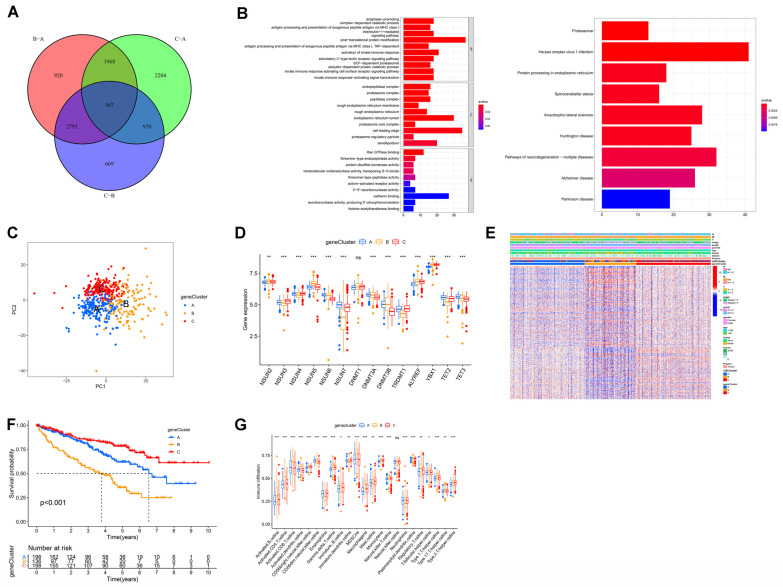
m5C gene clusters and corresponding biological features in ccRCC: (**A**) 567 m5C phenotype-related DEGs shown in Venn diagram; (**B**) Gene Ontology (GO, left) and Kyoto Encyclopedia of Genes and Genomes (KEGG, right) enrichment analyses of 567 DEGs. (**C**) PCA for transcriptome profiles of three m5C gene clusters. (**D**) Differences in expression of 14 m5C regulators in three m5C gene clusters. (**E**) Unsupervised clustering of overlapping m5C phenotype-related genes in TCGA to classify patients into different gene clusters, called m5C gene cluster A–C. (**F**) Kaplan–Meier overall survival (OS) curves for patients in three gene clusters. (**G**) Abundance of each TME-infiltrating cell in three m5C gene clusters. Adjusted *p*-values are shown as: ns, not significant; * *p* < 0.05; ** *p* < 0.01; *** *p* < 0.001; ns is not significant.

To further characterize the genetic profile of the different m5C clusters, we first used the “limma” package to screen for 567 DEGs relevant to the m5C phenotypes (Figure 4A). We performed KEGG and GO enrichment analyses for identifying the bio-processes in which these genes are involved. As shown in Figure 4B, these genes were enriched in several immune activation-related pathways, including activation of innate immune response and innate immune response-activating signal transduction, which confirmed that m5C is essential in regulating the ccRCC TME and immune activation. Subsequently, the following univariate COX analysis of these 567 genes, we screened 375 genes associated with prognosis for unsupervised cluster analysis, and we classified patients into three distinct m5C gene clusters, termed m5C gene clusters A–C (Figure 4C and Figure S2B). We analyzed the expression of 14 genes in these gene clusters and revealed that gene expression between the three gene clusters remained largely consistent with the previous m5C clusters (Figure 2C and Figure 4D). Additionally, the clinical characteristics and prognostic analyses of the three gene clusters were in line with the results of previous m5C clusters (Figure 4E,F). The above findings illustrated that m5C methylation exists in the three different modification patterns in ccRCC. Subsequently, we analyzed the composition of immune cells in the three m5C gene clusters. The ssGSEA results showed that the three isoforms were statistically significant in 22 cells (*p* < 0.05, Figure 4G). m5C methylation and its associated genes are crucial in the ccRCC TME and immune cell infiltration. 

### 3.5. Construction of m5Cscore and Its Clinical and Immunological Characteristics

**Figure 5:**
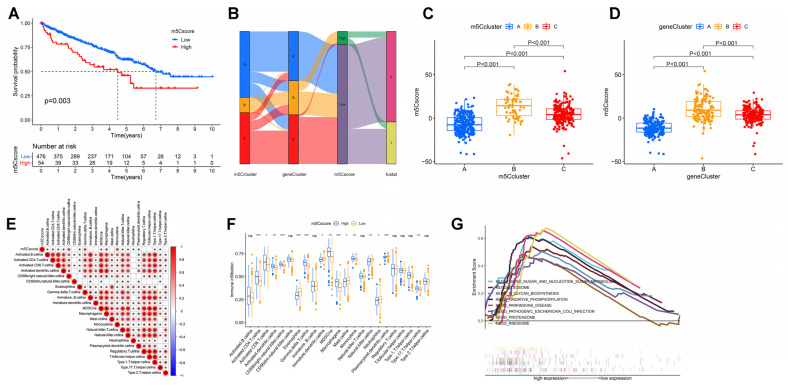
Construction of m5C signatures. (**A**) Kaplan–Meier survival analyses for low and high m5Cscore patient groups. (**B**) Alluvial diagram showing changes in m5Cclusters, gene cluster, m5Cscore, and patient survival status. (**C**,**D**) Differences in m5Cscore among m5C clusters (**C**) and three gene clusters (**D**). (**E**) Correlations between m5Cscore and known immune cells using Spearman analysis. (**F**) Abundance of each TME infiltrating cell in low- and high-m5Cscore group. (**G**) Enrichment plots show lysosome, N-glycan biosynthesis, ribosome, proteasome, and oxidative phosphorylation in low-m5Cscore subgroup. * *p* < 0.05; ** *p* < 0.01; *** *p* < 0.001; ns is not significant.

We observed individual heterogeneity in the m5C modifications, and this conclusion was based on a population of ccRCC patients where we failed to accurately predict the m5C modification profile of individual ccRCC patients. Therefore, based on m5C-associated DEGs, we developed a scoring system to quantify individual ccRCC patients’ m5C modification patterns, called the m5Cscore. We designed the “survminer” package for defining the optimal threshold for the m5Cscore and categorizing patients into high and low m5Cscore groups. Patients with low m5Cscore exhibited relatively better survival outcomes (Figure 5A). Changes in individual ccRCC patients’ attributes were visualized by alluvial diagram (Figure 5B). Subsequently, we used the Kruskal-Wallis test for analyzing the variations in the m5Cscore of the different m5C and gene clusters. As shown in Figure 5C,D, the median m5Cscore was the highest in cluster B and gene cluster B, and the lowest in cluster A and gene cluster A. In combination with the previous analysis, we thought that the worst prognosis for subgroup B may be related to its high m5Cscore (Figure 2D and Figure 4F). However, for subgroups A and C, the prognosis did not match their intra-score, probably due to the uneven distribution of patients between the two groups. 

Next, we further explored the value of the m5Cscore in predicting survival. The stratified prognostic analysis of patients by different clinical features revealed that low m5Cscore groups always displayed a favorable survival benefit (Figure S3A). In addition, we found a significantly lower proportion of patients with advanced tumors and the occurrence of metastases in the low m5Cscore group (Figure S3B) these patients also had a lower m5Cscore (*p* < 0.05, Figure S3C). Overall, the m5Cscore was associated with patient prognosis, with higher m5Cscores associated with poorer patient prognosis. This implied a more favorable prognostic outlook for the low-score group characterized by the m5C cluster C modification pattern and immune activation phenotype. These results indicated that the m5Cscore might be applied for assessing many ccRCC clinical features, including stage, grade, metastasis, and survival status.

After determining the prognostic value of the m5Cscore, we analyzed the immunological profile of the m5Cscore. First, we investigated the relationship between immune cells and the m5Cscore (Figure 5E and Figure S4A) and found that resting-memory CD4+ T cells, M1 macrophages, resting mast cells, monocytes, and resting NK cells decreased with increasing m5Cscore, with a negative correlation. Conversely, folic helper T cells, regulatory T cells, and M0 macrophages positively correlated with the m5Cscore, and cellular infiltration increased with increasing score. Following this, we estimated the TME immune infiltration in high- and low-m5Cscore groups. Figure 5F shows that adaptive immune cells, including CD4+ T, CD8+ T, and B cells, were predominantly increased in the high-m5Cscore subgroup. In contrast, the lo- m5Cscore subgroup had more intrinsic immune cells, such as neutrophils, plasmacytoid dendritic cells, and NK cells. Again, we applied the ESTIMATE algorithm and noted that the stromal score, which can also be considered as stromal cell infiltration in tumor tissue, was slightly higher in the low-m5Cscore group, whereas the opposite was true for immune score, although the infiltrative component in the TME did not significantly differ between the two groups (Figure S4B). Moreover, the results of GSEA uncovered that the high-m5Cscore group showed enrichment in many metabolic pathways and pathways related to genetic information processing, including ribosome, lysosome, and oxidative phosphorylation signaling. This result also explained the poor prognosis of the high-score group from an additional perspective (Figure 5G).

### 3.6. Correlation between m5C Modifications and Tumor Mutational Burden

**Figure 6:**
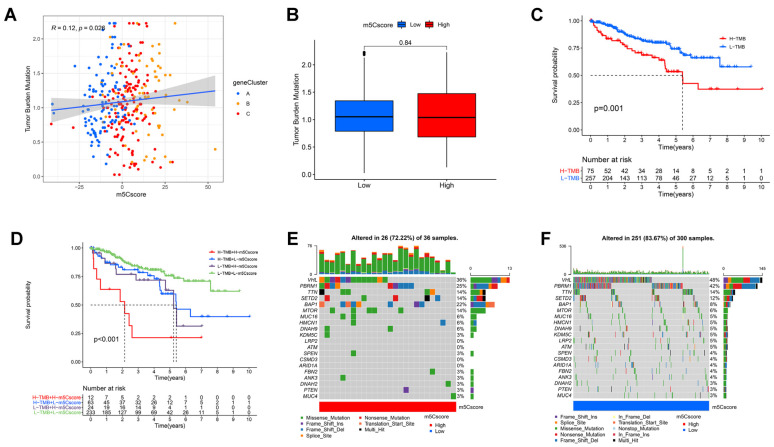
Correlation between m5Cscore and somatic variants. (**A**) Scatterplots depicting positive correlation between m5Cscore and TMB. (**B**) TMB difference in high- and low-m5Cscore subgroups (Wilcoxon test). (**C**) Kaplan-Meier curves for high- and low-TMB groups of ccRCC patients. (**D**) Kaplan–Meier curves for patients stratified by both TMB and ICI scores. (**E**,**F**) oncoPrint was constructed using high m5Cscores on left (**E**) and low m5Cscores on right (**F**).

From the above immunoassay, we found remarkable variations in immune infiltration between two groups. Immune cell infiltration in ccRCC correlated with tumor mutational burden (TMB), with the high TMB group having lower levels of infiltration than the low TMB group [39]. This implied that tumor-loading mutations (TMB) may predict patient prognosis and determine the individual response to cancer immunotherapy [40]. Considering the clinical value of TMB, we first explored the relevance of the m5Cscore to TMB. The results of correlation analysis confirmed a significant positive correlation between the m5Cscore and TMB (Spearman’s coefficient: R = 0.12, *p* = 0.026; Figure 6A). However, we observed no statistical difference in TMB levels between two groups (Figure 6B). We then divided patients into high-TMB and low-TMB groups. A better OS for the low TMB patients could be observed in Figure 6C (*p* = 0.001). Considering the contraindicated prognostic value of the TMB and m5Cscore, we further assessed the synergistic impact of both in ccRCC prediction stratification. The results of stratification analysis showed that TMB and m5Cscore did not interfere with each other’s survival predictions based on each other. The m5Cscore subtype showed a significant prognostic difference between two TMB subgroups (*p* < 0.001, Figure 6D). Overall, these results proved that the m5Cscore can be considered a TMB-independent predictor. Moreover, we used the “maftools” package to compare the distribution of somatic variants between two groups. Figure 6E demonstrates that the low-score group had a wider range of TMB than the high-m5Cscore group. These results yielded novel insights into the mechanisms of oncogene mutations and implicitly validated m5Cscore’s value in forecasting the effects of immunotherapy.

### 3.7. Value of m5Cscore in Predicting Immunotherapy Efficacy

Currently, immunotherapy, especially ICIs, has become key in oncology treatment [41]. However, some patients still fail to respond to immunotherapy, which somewhat restricts the use of ICIs. Therefore, Charoentong et al. established a quantitative scoring scheme named the immunophenotype score (IPS) to identify the factors affecting tumor immunogenicity. Among others, the IPS is an excellent predictor of anti-PD-1 and anti-CTLA-4 antibody responses [29]. In our study, we used the IPS to more visually assess the value of the m5Cscore in predicting immunotherapy. We applied four categories (IPS-CTLA4-neg-PD-1-neg, IPS-CTLA4-neg-PD-1-pos, IPS-CTLA4-pos-PD-1-neg, and IPS-CTLA4-pos-PD-1-pos) for evaluating the likelihood of patients receiving different treatment ICI regimens. The results demonstrated significantly higher scores in the low-m5Cscore group (Figure S5A, IPS-CTLA4-pos-PD-1-neg, *p* = 1.4 × 10^−6^; IPS-CTLA4-neg-PD-1-pos, *p* = 0.025). The low-m5Cscore group had a higher IPS, suggesting that a more immunogenic phenotype, and ICIs might be more effective. Moreover, we investigated some common immune molecules expressed in different m5Cscore groups. We noticed that almost all genes, except CD40, were expressed at higher levels in the low-m5Cscore group (Figure S5B). This was also probably a main driver of the higher survival rate in the low-m5Cscore group (Figure 5A). The above results illustrated that quantifying m5C modification patterns may serve as a promising and consistent biomarker for immunotherapeutic assessment.

### 3.8. Validation of m5Cscore Performance in Predicting Prognosis

Using the previously obtained 375 m5C prognosis-related DEGs, we recreated the m5C gene signature and calculated the m5Cscore for the data four databases: TCGA-KICH, TCGA-KIRP, TCGA-LIHC, and TCGA-OV. Figure S6A–D show the efficacy of the m5Cscore in predicting the OS for each of the four cancers. Consistent with ccRCC, the low m5Cscore group showed higher prognostic value in KICH and LIHC (Figure S6A,C). However, in KIRP and OV, the performance of the m5Cscore in predicting patient survival was not statistically significant (Figure S6B,D). This suggested that m5C methylation may also be involved in the development of KICH and LIHC.

## 4. Discussion

m5C modifications are critical in inflammation, infection, and tumors that rely on the interaction of multiple m5C regulators [42,43]. Most researchers have focused on one regulator or only on the prognosis of cancer patients [44,45]; therefore, the value of the entire m5C modifications in regulating tumor prognosis as well as immune microenvironmental infiltration must be further explored. Currently, m6A modifications have been well-explored in tumor immune infiltration in renal cancer [46]. In this study, we highlighted m5C modification in ccRCC TME cell infiltration to enhance our comprehension of the antitumor immune response and to allow for the design of targeted immunotherapeutic regimens. 

In this study, based on 14 m5C regulators, we ascertained three distinct m5C modification patterns, with distributions exhibiting different immune cell infiltration characteristics. The ESTIMATE results indicated a significant activation of the immune component in cluster B. In combination with immune cell infiltration characteristics, we suggest that cluster B corresponded to immune-excluded phenotype and contained a large number of immune cells, but these cells were present in the tumor stroma and failed to penetrate the parenchyma, thus limiting the immunological effect of the immune cells. Cluster A corresponded to an immune-desert phenotype and was mainly immunosuppressive, linked to immune tolerance and a lack of T cell activation. Cluster C corresponded to immune-inflamed phenotype and was characterized by many adaptive immune cells with the infiltration of myeloid cells and monocytes, thus activating the immune response. This confirmed the validity of m5C modifications in the classification of immune phenotypes. Furthermore, we found the differential genes between different m5C modification patterns to be significantly associated with the biological pathways associated with immune activation. These genes were considered to be m5C-related signature genes. Based on these genes, we also defined three genomic clusters. The clinical and immunological characteristics between these gene clusters were similar to those of the m5C clusters described above, which also flanked the essential function of m5C modification, constructing a diverse TME environment. Thus, a thorough evaluation of m5C modification patterns will enhance our understanding of TME cell infiltration characteristics and can help with improving the application of precisely targeted, personalized therapies for ccRCC in the future.

Given the interindividual variation in m5C modifications, we applied a scoring system to precisely evaluate m5C modification patterns in individual ccRCC patients, which we called the m5Cscore. We concluded that the higher the m5Cscore, the worse the patient prognosis. The patients in m5C cluster B, featuring the immune-excluded phenotype, received the highest m5Cscore and the worst prognosis. The reason why m5C cluster C had the best prognosis but not the lowest m5Cscore may be due to the uneven number of people in two m5Cscore subgroups. These results were also well-verified in the m5C gene cluster. Overall, we concluded that the higher the m5Cscore, the worse the prognosis of the patient. This suggested that the m5Cscore can be used as a credible predictive indicator for ccRCC and can contribute to a comprehensive assessment of individual m5C modification patterns.

Tumor immunotherapy, in which tumor immune checkpoint inhibitors (ICIs) are the most clinically well-established and well-researched, is the most widely used [47,48]. ICIs are mainly dominated by CTLA-4 and PD-1 inhibitors (PD-1/PD-L1 inhibitors) [49]. Compared with chemotherapeutic agents, these drugs are durable, broad-spectrum, and have low toxicity [50,51]. Nevertheless, some patients with ccRCC remain unresponsive or develop resistance to immunotherapy [52]. Therefore, we need to find a reliable evaluation metric that accurately predicts the efficacy of ICIs. CD8+ T-cell infiltration coupled with the presence of nonsynonymous mutations can significantly enhance patient response to anti-PD-1 therapy [53,54]. In this study, we confirmed the prognostic value of the m5Cscore in both anti-PD-1 and CTLA-4 immunotherapy cohorts, with patients with low m5Cscores being more likely to receive ICI therapy and having better treatment outcomes. Thus, we not only demonstrated that m5C modification patterns can shape different aspects of the immune TME landscape but also largely influence ICI treatment outcomes. 

Within clinical applications, the m5Cscore serves as a comprehensive assessment of individual patients’ m5C modification patterns and corresponding immune cell infiltration features to facilitate the judgement of tumor immunophenotypes and guide the more efficient clinical administration of drugs. Currently, several researchers have identified RNA m5C methylation as a promising new target for tumor therapy. Bioinformatics studies showed that m5C regulators can be used to predict the prognosis of lung adenocarcinoma [55], head and neck squamous cell carcinoma [56], and hepatocellular carcinoma [57]. In our study, we not only demonstrated that the m5Cscore allows for the assessment of patients’ clinicopathological signatures: we also validated its value in predicting the clinical response of patients to anti-PD- 1/CTLA-4 therapy. RNA m5C methylation may be a new target and biomarker for the treatment and diagnosis of ccRCC. We are therefore able to provide clinicians novel thoughts on patient staging and individualized treatment. 

However, the study still contained shortcomings. All of our studies were based on the use of bioinformatics to examine database information, and clinical cases were missing to further determine the correlation between m5C modification and ccRCC prognosis and immune infiltration. Given the apparently uneven distribution of numbers in some of the subgroups in the study, some of the results need to be further validated through prospective clinical cohorts. In future work, we will conduct further clinical and basic trials to validate the results. 

## 5. Conclusions

In conclusion, we demonstrated the mechanism by which m5C methylation governs ccRCC TME infiltration. Interindividual differences in m5C methylation were one of the main reasons for the heterogeneity of tumor immune infiltration. Thorough evaluation of tumor m5C modification patterns might enhance our knowledge of the TME and immune infiltration and be used to guide more effective personalized immunotherapeutic approaches in the clinic.

## Data Availability

The data used in this study can find at the GDC website (https://portal.gdc.cancer.gov/ (accessed on 6 December 2020)) and the ICGC database (https://icgc.org/ (accessed on 6 December 2020)).

## Supplementary Materials

The following supporting information can be downloaded at: https://www.mdpi.com/article/10.3390/curroncol30010044/s1, Table S1. Differences in expression of 14 m5C regulators in ccRCC in TCGA and ICGC databases. Table S2. The prognostic analyses for 14 m5C regulators using a univariate Cox regression model. Figure S1. The differential expression of m5C regulators at the protein level and its relevance to the prognosis of ccRCC patients. Figure S2. Unsupervised clustering of 567 m5C phenotype-associated genes in the TCGA cohort and consensus matrix, k = 3. Figure S3. Differences in prognostic performance and expression of m5Cscore across clinical subgroups. Figure S4. Correlation of m5Cscore expression with immune cells and the results of the ESTIMATE algorithm. Figure S5. m5C modification patterns in the role of ccRCC clinical therapies. Figure S6. Validation of prognostic performance of m5Cscore in KICH, KIRP, LIHC and OV.
