# Supplementary material for: 5-Methylcytosine (m5C) Modification Patterns and Tumor Immune Infiltration Characteristics in Clear Cell Renal Cell Carcinoma
Source: Curr Oncol. 2022 Dec 31;30(1):559–74. doi: 10.3390/curroncol30010044 (PMC9857358; doi:10.3390/curroncol30010044)
Supplement: Supplementary file 1 [file curroncol-30-00044-s001.zip › curroncol-2107371-supplementary.pdf]

Article

5-Methylcytosine (m5C) Modification Patterns and Tumor Immune Infiltration Characteristics in Clear Cell Renal Cell Carcinoma

Can Chen, Lin-Yuan Chen, Jie-Xin Zhang and Hua-Guo Xu

Supplemental Materials

Table of Contents

Table S1. Differences in expression of 14 m5C regulators in ccRCC in TCGA and ICGC databases.

Table S2. The prognostic analyses for 14 m5C regulators using a univariate Cox regression model.

Figure S1. The differential expression of m5C regulators at the protein level and its relevance to the prognosis of ccRCC patients.

Figure S2. Unsupervised clustering of 567 m5C phenotype-associated genes in the TCGA cohort and consensus matrix, k = 3.

Figure S3. Differences in prognostic performance and expression of m5Cscore across clinical subgroups.

Figure S4. Correlation of m5Cscore expression with immune cells and the results of the ESTIMATE algorithm.

Figure S5. m5C modification patterns in the role of ccRCC clinical therapies.

Figure S6. Validation of prognostic performance of m5Cscore in KICH, KIRP, LIHC and OV.

Table S1. Differences in expression of 14 m5C regulators in ccRCC in TCGA and ICGC databases.

	TCGA			ICGC		
**gene**	**Normal**	**ccRCC**	**pValue**	**Normal**	**ccRCC**	**pValue**
NSUN2	3.135	3.435	<0.001	5.790	6.849	<0.001
NSUN3	1.472	1.400	0.025	4.215	4.215	0.790
NSUN4	2.186	2.009	<0.001	5.049	4.837	<0.001
NSUN5	2.336	2.478	0.003	5.198	5.774	0.011
NSUN6	1.501	1.748	<0.001	5.060	5.126	0.094
NSUN7	1.730	1.074	<0.001	4.481	3.401	<0.001
DNMT1	2.083	2.360	<0.001	4.636	5.508	<0.001
DNMT3A	1.609	1.799	<0.001	4.353	4.777	<0.001
DNMT3B	0.726	0.947	<0.001	3.488	3.746	0.959
TRDMT1	0.887	0.879	0.781	3.518	3.556	0.797
ALYREF	3.995	4.197	<0.001	7.690	8.712	0.002
YBX1	7.296	7.334	0.043	43.248	43.605	0.495
TET2	1.433	1.513	0.035	4.127	4.320	0.017
TET3	0.981	1.571	<0.001	3.915	4.222	<0.001

Table S2. the prognostic analyses for 14 m5C regulators using a univariate Cox regression model.

**id**	**HR**	**HR.95L**	**HR.95H**	**pvalue**
NSUN2	1.036	0.902	1.191	0.616
NSUN3	0.695	0.475	1.018	0.062
NSUN4	0.738	0.508	1.073	0.111
NSUN5	1.328	1.165	1.515	<0.001

NSUN6	1.155	1.053	1.268	0.002
NSUN7	0.806	0.700	0.928	0.003
DNMT1	0.968	0.803	1.165	0.728
DNMT3A	1.371	0.971	1.937	0.073
DNMT3B	1.325	1.201	1.462	<0.001
TRDMT1	0.593	0.423	0.832	0.002
ALYREF	1.050	0.951	1.159	0.338
YBX1	0.994	0.983	1.006	0.320
TET2	0.630	0.481	0.825	0.001
TET3	1.094	0.774	1.544	0.611

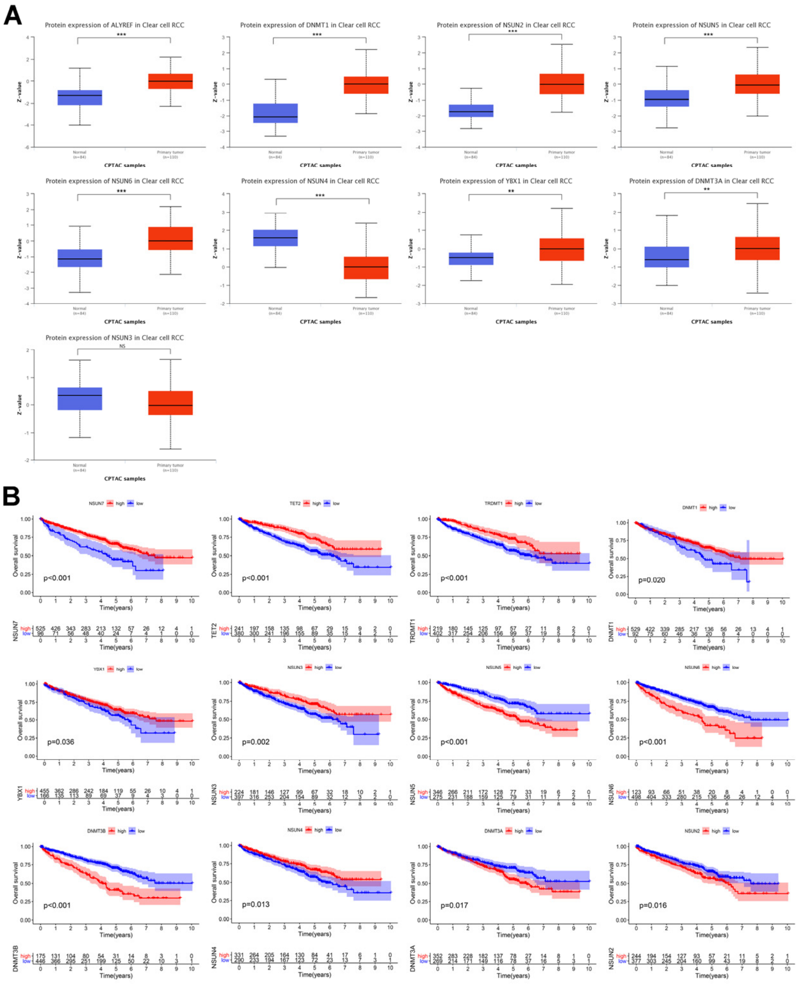

Figure S1. (A) Differential expression of the m5C methylation regulators at the protein level. (**B**) Kaplan-Meier curves demonstrate the prognostic relevance of m5C methylation regulators expression to ccRCC patients.

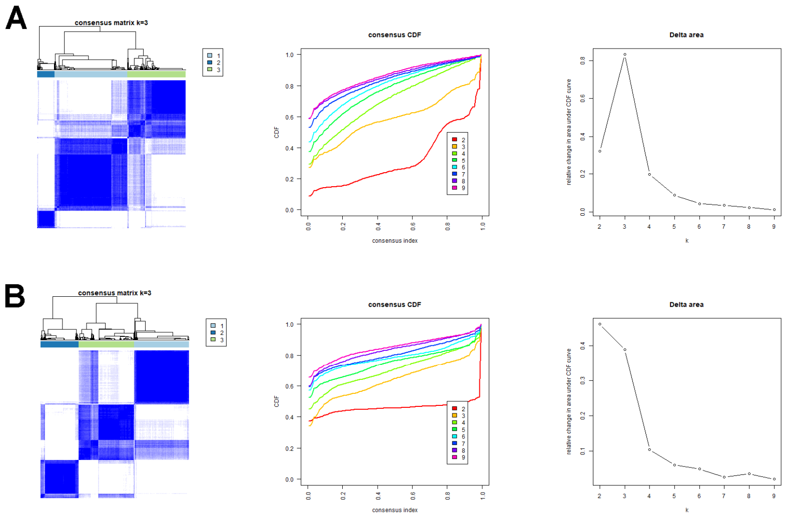

Figure S2. **(A)** Heat map of the consensus matrix for the ccRCC sample at k = 3 (left). Cumulative distribution function curves for unsupervised clustering of 14 m5C regulators, k = 2-9 (middle). Relative change in area under the CDF curve for unsupervised clustering of 14 m5C regulators, k = 2-9 (right). (**B)** Heat map of the consensus matrix for k = 3 (left). Cumulative distribution function curves for unsupervised clustering of 567 m5C phenotype-related genes in ccRCC cohort, k = 2-9(middle). Relative change in area under the CDF curve for unsupervised clustering of 567 m5C phenotype-associated genes, k = 2-9 (right).

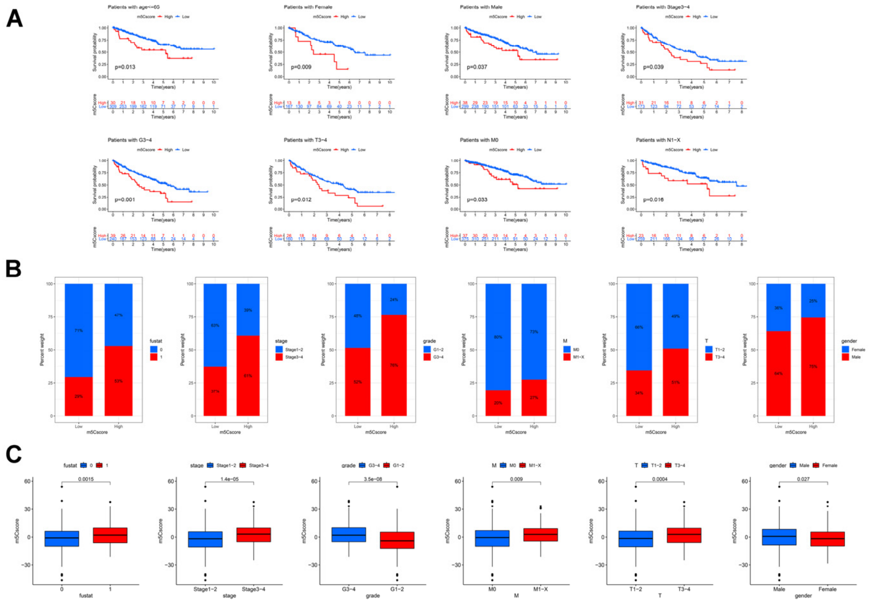

Figure S3. **(A)** Kaplan-Meier curves depicted the survival difference between low and high m5Cscore in the stratified analysis of ccRCC patients, including age, gender, grade, stage and TMN stage. **(B)** The proportion of patient survival status, stage, grade, M, N and gender in high- and low-m5Cscore groups. (**C)** Boxplots for m5Cscore between different characteristics of ccRCC patients, including patient survival status, stage, grade, M, N and gender.

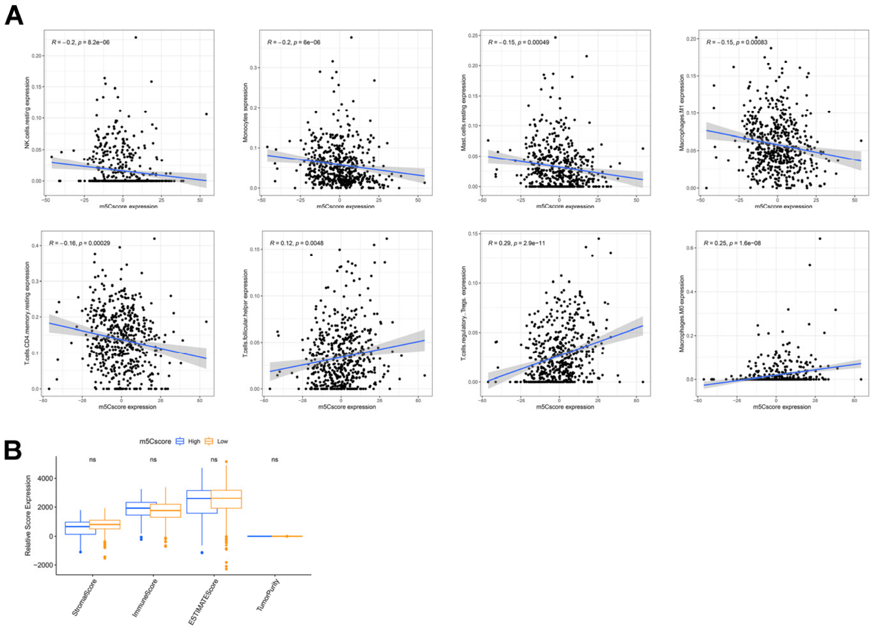

Figure S4. (A) The correlation between m5Cscore and several immune cell, including resting NK cell, monocytes, resting mast cell, macrophages M1, resting memory CD4+ T cells, follicular helper T cells, Tregs and macrophages M0. **(B)** The box plot indicated difference in immune scores, stromal scores estimate score and tumor purity between low- and high-m5Cscore group.

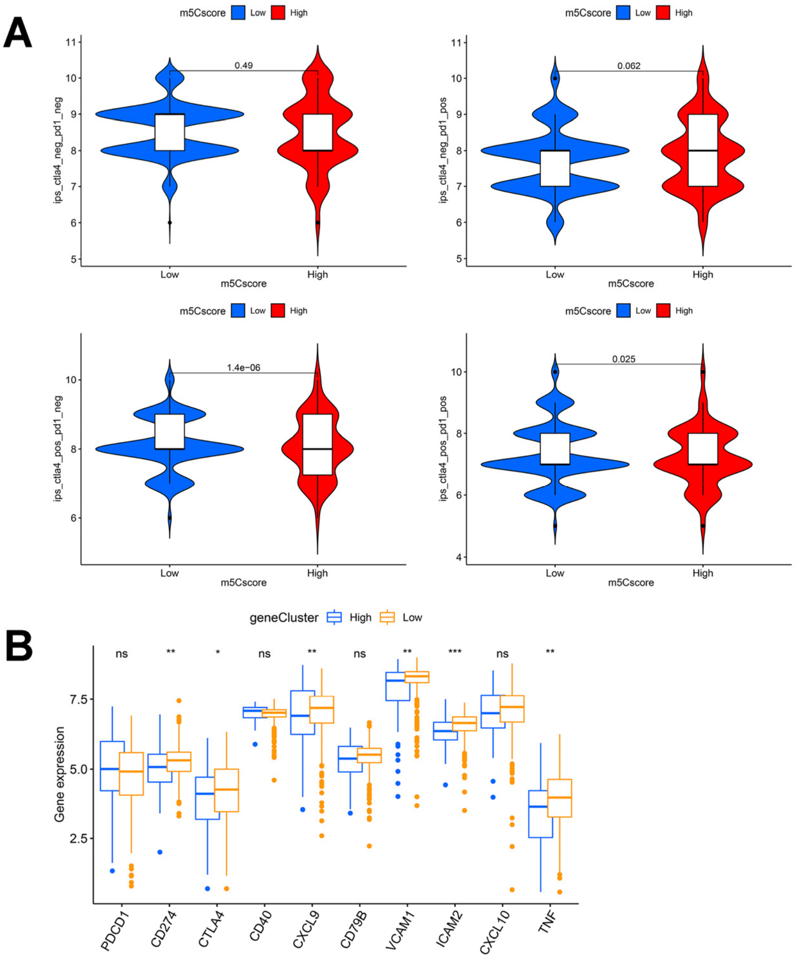

Figure S5. (A) The association between IPS and immune checkpoints in ccRCC patients. **(B)** Immune-relevant genes (PDCD1, CD274, CTLA-4, CD40, CXCL9, CD79B, VCAM1, ICAM1, CXCL10 and TNF) expressed in high and low m5Cscore subgroups.

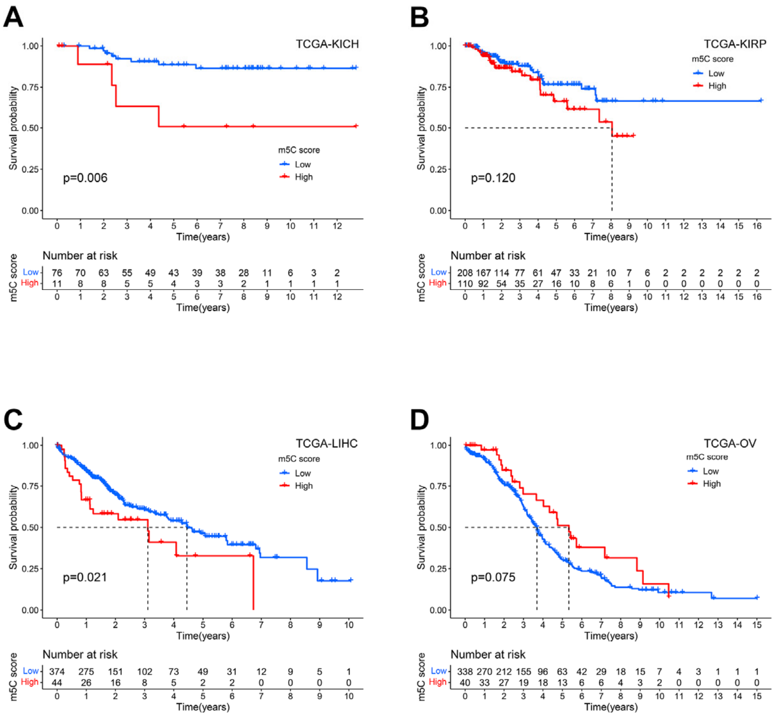

Figure S6. (A) Kaplan-Meier curves predict the overall survival rate of m5Ccsore in KICH. **(B)** Kaplan-Meier curves predict the overall survival rate of m5Ccsore in KIRP. **(C)** Kaplan-Meier curves predict the overall survival rate of m5Ccsore in LIHC. **(D)** Kaplan-Meier curves predict the overall survival rate of m5Ccsore in OV.
